# Carrier Transport in Colloidal Quantum Dot Intermediate Band Solar Cell Materials Using Network Science

**DOI:** 10.3390/ijms24043797

**Published:** 2023-02-14

**Authors:** Lucas Cuadra, Sancho Salcedo-Sanz, José Carlos Nieto-Borge

**Affiliations:** 1 Department of Signal Processing and Communications, University of Alcalá, 28805 Madrid, Spain; 2 Department of Physics and Mathematics, University of Alcalá, 28805 Madrid, Spain

**Keywords:** colloidal quantum dots, hopping transport, intermediate band solar cell, space-energy embedded networks, complex networks

## Abstract

Colloidal quantum dots (CQDs) have been proposed to obtain intermediate band (IB) materials. The IB solar cell can absorb sub-band-gap photons via an isolated IB within the gap, generating extra electron-hole pairs that increase the current without degrading the voltage, as has been demonstrated experimentally for real cells. In this paper, we model the electron hopping transport (HT) as a network embedded in space and energy so that a node represents the first excited electron state localized in a CQD while a link encodes the Miller–Abrahams (MA) hopping rate for the electron to hop from one node (=state) to another, forming an “electron-HT network”. Similarly, we model the hole-HT system as a network so that a node encodes the first hole state localized in a CQD while a link represents the MA hopping rate for the hole to hop between nodes, leading to a “hole-HT network”. The associated network Laplacian matrices allow for studying carrier dynamics in both networks. Our simulations suggest that reducing both the carrier effective mass in the ligand and the inter-dot distance increases HT efficiency. We have found a design constraint: It is necessary for the average barrier height to be larger than the energetic disorder to not degrade intra-band absorption.

## 1. Introduction

Quantum dots (QDs) are zero-dimensional nano-structures that confine carriers in the three directions of space, showing thus atomic-like features (“artificial atoms”), such as discrete energy levels, well separated by zero density of states (DOS) [1,2,3]. In particular, colloidal quantum dots (CQDs), sometimes called colloidal nanocrystals (NCs), can be synthesized via low-cost sequences [4] of wet chemical processes that produce NCs dispersed in a solvent. These chemical methods not only allows for controlling the dot size (and thus, the energy levels) but also their homogeneous dispersibility in the solvent, and what is very important, performing “ligand engineering” [5,6,7]. This aims at selecting the shell of surface ligands which assists in [6]: (a) Stabilizing the NC in different organic solvents; (b) Passivating the defects on the NC surface that act as non-radiative recombination centers [8,9,10,11,12], reducing the traps density [7] and increasing the carrier lifetimes in solar cells; (c) Exchanging ligands for others to control the inter-dot distance (and thus the coupling between dots); (d) Exchanging long ligands for shorter ones that work as electronic bridges between NCs to increase carrier extraction [13,14,15]. (e) Establishing their *p*-type or *n*-type nature [16]; (f) Controlling the energy of the band edges in conventional II-VI and IV-VI CQDs [17,18,19].

As can be inferred from the aforementioned features (a)–(f), ligand engineering is based on a great number of experimental approaches to enhance both the optoelectronic properties and the stability of CQDs [6,20,21,22]. As emphasized in [23], ligand engineering is likely the most critical step for improving CQD-based devices. This is just the reason why big-data-driven Machine Learning [24] has been recently proposed as a powerful tool to suggest feasible ligands that can guide and accelerate the experimental work [6,23,25,26]. Because of all the aforementioned beneficial properties, CQDs are considered as the “building blocks” [27,28] for manufacturing an important variety of opto-electronics devices [1,29,30,31].

**Figure 1:**
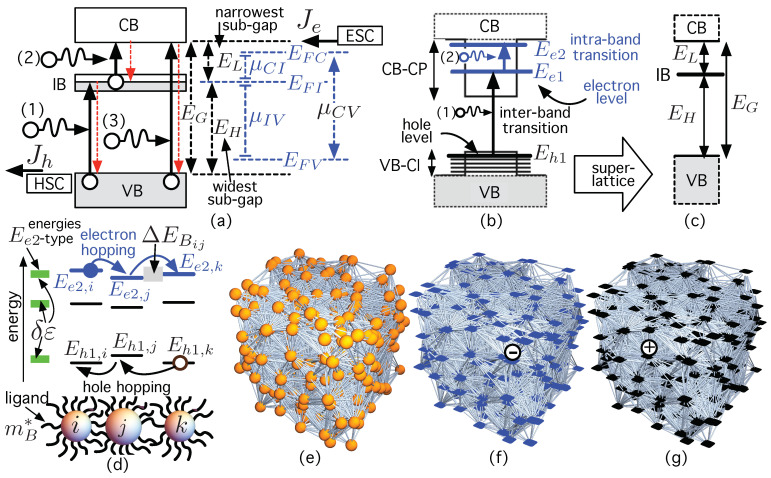
(**a**) Simplified illustration of the gaps and operation of an IBSC. (**b**) Single QD showing intra-band transition ($E_{e1}\to E_{e2}$) and inter-band transition ($E_{h1}\to E_{e1}$). (**c**) Resulting IB material. (**d**) Disorder causes electron hopping between localized states with different energy: $E_{e2,i}\to E_{e2,j}$ at QDs *i* and *j*. The same argument is applied to the case of hole hopping. δε and $\Delta E_{B_{ij}}$ stand for the energetic disorder and the barrier height between dots *i* and *j*. (**e**) CQD-network. (**f**) Network modeling electron hopping between $E_{e2}$-type levels shown in (**d**). (**g**) Hole hopping network involving $E_{h1}$-type levels shown in (**d**). See the main text for further details.

In the particular case of colloidal solar cells, [31,32,33,34,35,36], CQDs have also been proposed as a feasible technology to put into practice the concept of Intermediate Band Solar Cell (IBSC) in several works [37,38,39,40,41,42]. The IBSC was proposed by A. Luque and A. Martí [43] to overcome one of the limitations of the single-gap solar cell (SGSC) [44,45,46]: those photons whose energy is lower than the semiconductor gap, $E_{G}$, (called “sub-band-gap photons”, $E<E_{G}$) are wasted because they have not enough energy to excite electrons through the forbidden gap $E_{G}$, and thus cannot generate electron-hole pairs. In contrast, as shown in Figure 1a, the IBSC can absorb the sub-band-gap photons (1) and (2) via an isolated intermediate band (IB), increasing the current without degrading the voltage *V*, as has been demonstrated experimentally for real cells at room temperature [47]. The absorption of the sub-band-gap photon (1) pumps an electron from the valance band (VB) to the IB while that of the photon (2) excites another electron from the IB to the conduction band (CB) [43]. This extra, two-step, “two-photon photo-current” (TPPC)—the first IBSC operating principle—is added to the conventional one caused by photons like (3) with $E>E_{G}$. This higher current is injected without degrading its output voltage, which is only limited by $E_{G}$, and not by any of the two sub-gaps ($E_{L}$ or $E_{H}$) the IB divides $E_{G}$. This, called “voltage preservation” (VP), is the second operating principle the IBSC is based on.

Figure 1a also illustrates some important concepts for the purpose of our paper. The first one is that, when working, the IBSC has three quasi-Fermi levels, $E_{FC}$, $E_{FI}$, and $E_{FV}$, for describing the electron concentrations in the three bands involved. That is, there must be three electron gases separated by zero DOS. The second key concept is that the IB must be partially filled with electrons (“metallic nature”) [48] aiming to have both empty states (to accommodate electrons excited from the VB) and to have electrons (to be pumped into the CB) [49,50]. Finally, the IB absorbing material is sandwiched between an electron selective contact (ESC) and a hole selective contact (HSC) [49,50]. These allow for both isolating the IB and injecting the increased current density—electrons from the CB ($J_{e}$) and holes from the VB ($J_{h}$)—at a high voltage *V*. This corresponds to the separation of the quasi-Fermi levels for the majority carrier in the selective contacts, $eV=E_{F,e}-E_{F,h}\equiv E_{FC}-E_{FV}\equiv \mu_{CV}=\mu_{CI}+\mu_{IV}$, *e* being the electron charge, and $\mu_{YX}$ the chemical potential of the luminescent radiation between the Y and X bands [49,50]. Its limiting efficiency—using detailed balance concepts in the radiative limit and without violating the Second Law of Thermodynamics [51,52]—has been found to be 63.2% (fully concentrated sunlight) [43], much higher that that of the SGSC (40.7% [53]). The two operating principles of the IBSC model (TPPC and VP) have been experimentally proved [47,54,55,56,57,58,59,60,61] in devices based on stacks of layers of self-assembled quantum dots (SAQDs), using epitaxial techniques such as molecular beam epitaxy (MBE) [62]. Figure 1b shows a single “epitaxial” quantum dot (EQD) with its corresponding CB confinement potential (CB-CP) and BV confinement potential (VB-CP). $E_{e1}$ is the ground electron level, while $E_{e2}$ is the first excited level in the CB-CP. $E_{h1}$ is the first hole level in the CV-CP. As shown in Figure 1c, the IB should ideally arise from the intermediate level $E_{e1}$ in a super-lattice of QDs [63]. Based on a super-lattice of InN/In${}_{x}$Ga${}_{1-x}$N QDs, the influence of the internal electric field produced by the polarization in the active QD region has been studied in [64].

However, there are still two problems that need to be addressed. The first one is that the separation between the ground level $E_{e1}$ and the first excited level $E_{e2}$ in EQDs is not large enough to prevent electron thermalization [65,66] at room temperature, degrading thus the voltage. The second problem is that photon absorptions causing transitions via the IB are too small [67,68]. This makes the sub-band-gap photocurrent be orders of magnitude smaller than the conventional one [69]. One of the possible causes of this weak absorption could be the low EQD volumetric density (∼$10^{15}$–$10^{16}$ cm${}^{-3}$) [70]. In this respect, CQDs could help overcome these limitations. On the one hand, the phonon bottleneck effect recently observed in doped CQDs [71,72] could prevent electron thermalization, the first of the remaining issues. On the other hand, CQDs can be densely packed (∼$10^{19}$–$10^{20}$ cm${}^{-3}$) and also heavily n−doped, leading to a high absorption coefficient (∼$10^{4}$ cm${}^{-1}$) for the intra-band transition $E_{e1}\to E_{e2}$ [70], similar to that of inter-band transitions $E_{h1}\to E_{e1}$. (See Figure 1b). This could help overcome the second-mentioned problem.

Nevertheless, not everything is positive because the CQD approach suffers from an important problem: CQD ensembles have much slower mobilities μ than bulk crystalline semiconductors. Several works [13,73,74,75,76,77,78,79] claim that low mobilities (μ<1 cm${}^{2}$ V${}^{-1}$ s${}^{-1}$) are caused because the dominant carrier transport mechanism is the “carrier hopping” between localized states disordered in both space and energy. At present, even in the best CQD samples, the remaining standard deviation in the dot size and the chemical variation on the CQD surface or in its ligand may introduce energetic disorder δε (green rectangles in Figure 1d). Disorder causes electron hopping between localized states with different energy: $E_{e2,i}\to E_{e2,j}$ (“$E_{e2}$-like” levels) at CQDs *i* and *j*. The same argument is applied to the case of hole hopping. In this respect, Figure 1d shows electron hopping ($E_{e2,i}\to E_{e2,j}\to E_{e2,k}$), and hole hopping ($E_{h1,k}\to E_{h1,j}\to E_{h1,i}$). There is some controversy about the type of conductivity that appears in highly mono-disperse (dot size standard deviation ∼3%), dense, close-packed, ordered CQD thin films [30,80,81], which should ideally lead to superlattices (SLs) [82,83,84]. In this case, some works suggest that the increased mobility, in the order of tens of cm${}^{2}$ V${}^{-1}$ s${}^{-1}$, is caused by a transition from hopping transport (HT) to band-like transport [80,85,86,87]. However, there is no consensus on whether this is truly the case or what the possible cause is.

The purpose of this work is to explore electron and hole HT in CQD-IB-materials— as shown in Figure 1e—using networks that represent electron-HT—Figure 1f—and hole-HT—Figure 1g—. Network Science (NS) involves a conceptual framework and a set of mathematical tools that help study different systems, which consist of a large number of interacting elements [88]. These complex systems can be represented using a network (or, mathematically, a graph) [89], with “nodes” (vertices) connected by “links” (edges). A node represents an interacting entity of a system (for instance, a server in a communication network), which is linked to others by exchanging information (in communication networks [90,91]), matter (sap in vascular networks in plants [92]), or energy (in a power grid [93]). By properly representing systems as networks, NS assists in understanding the underlying structure and the emergence of collective phenomena in very different complex systems [88,94,95,96], involving not only natural ones (the human brain [97], ecosystems [98], vascular networks [99], interstellar molecular complexity [100], or complex Earth systems [101]) but also engineering systems (power grids [93,102,103], the Internet [104], blockchain [105] or transportation networks [106]). More examples can be found in [88,95,107] and the references therein. Furthermore, NS math tools [107,108,109] also help analyze dynamic processes involving the spread of epidemics [110] such as COVID-19 [111], cascading failures in technological networks [112,113] or the spreading [114] and persistence of information, memes or ideas [115]. For deeper concepts, the interested reader is referred to [107]. While NS has been extensively applied to a wide variety of “macroscopic systems”, it has been used to a much lesser extent to explore “nano-systems”. The recent work [116] studies HT in organic disordered semiconductors as networks embedded in space and energy, on which carrier transport is modeled using continuous-time random walks (CTRW) [117]. Any localized quantum state is represented by a node, while carrier hopping between nodes is encoded by a link. Other organic solar cells with efficient charge transport and collection have been reported in [118,119]. Systems of QDs have also been modeled as networks in [120,121]. The first one focuses on representing a disordered ensemble of QDs (=nodes) as a spatial network with links given by the electron overlap integrals between the QDs. In the second example of the application of NS on QD systems [121], QDs have different sizes and energy levels. In both works [120,121], continuous-time quantum walks (CTQW)—quantum walks on continuous time, and discrete space [122]—has been used to study quantum transport (QT). Although without the energetic and spatial constraints of electrons in QDs, other earlier works have investigated QT in different networks, such as in regular lattices [117,123,124], branched structures [125,126], fractal patterns [127], Husimi cacti [128], Cayley trees [129], Apollonian networks [130], or start graphs [131,132]. See [117] for further details.

Specifically, in this paper, we model the electron-HT system as a network embedded in both space and energy so that a node represents a localized electron state with energy $E_{e2}$-type in a CQD while a link encodes the probability (or, equivalently, the Miller–Abrahams (MA) hopping rate [133]) for the electron to hop from one node (=state) to another, forming an “electron-HT network”, as schematically represented in Figure 1e. Similarly, we model the hole-HT system as a network embedded in space and energy so that a node encodes a localized hole state with energy $E_{h1}$-type in a CQD while a link represents the MA hopping rate for the hole to hop from one node (=state) to another, leading to a “hole-HT network” (Figure 1f). The associated network Laplacian matrices allow for studying carrier dynamics using edge-centric random walks [134], in which links are activated by the corresponding carrier hopping rates.

The rest of this paper is structured as follows. After presenting in Section 2 our model to define both electron-HT and hole-HT networks, Section 3 shows the results and the corresponding discussion. The main results are that a decrease in the carrier effective mass in the barrier/ligand ($m_{B}^{*}$ in Figure 1d) and/or a reduction of the inter-dot distance (i.e., a higher dot density) leads to an improvement in carrier HT efficiency in both networks. We have also found a design constraint: the mean value of the potential barrier height ($\bar{\Delta E_{B}}$) that carriers have to tunnel cannot be less than the energetic disorder δε (green rectangles in Figure 1d). If $\bar{\Delta E_{B}}$<δε then there will be CQDs whose $E_{e2}$-type levels would be in the continuum. This reduces sub-band-gap photon absorption because a transition between a localized state with energy $E_{e1}$ and an extended state is much less likely. Finally, Section 4 completes the paper with the main concussions.

## 2. Materials and Methods

### 2.1. Hypotheses

As mentioned, our purpose is to represent CQD materials as electron and hole networks on which to study carrier transport. Our model could be used to represent different CQD systems. An interesting example is a solution-processed material with PbS CQDs, disordered and densely dispersed in a CH${}_{3}$NH${}_{3}$PbBr${}_{3}$ perovskite matrix [41]. This material exhibits two-step photon absorption via an IB at room temperature [41]. Other examples of disordered CQD materials have been reported in [86], manufacturing a highly mono-disperse CdSe QD film with a short inorganic ligand that leads to crack-free, randomly close-packed QD thin-films, or in [135] with mono-disperse HgTe CQDs and a proper ligand-engineered that produces electron mobility of up to 18 cm${}^{2}$ V${}^{-1}$. Other approaches consider a CQD-solid made up of several dense, close-packed, *ordered* CQD thin films, leading to a volumetric dot density $N_{D}$. A good example is the formation of well-ordered square and honeycomb superlattices of CdSe QDs in a CdS matrix [82]. Conceptually, in the regime of strongly coupled CQDs, band-like transport may appear. In this case, the coupling between adjacent dots should produce delocalized states, similar to the coupling of individual atoms within a lattice, forming a band [84]. However, completely delocalized band-like structures are expected to be very difficult to achieve because even the smallest amount of size fluctuation could avoid delocalization [84].

As we will show later on, our model allows for locating the CQD in space, either in an ordered or disordered way, and computing the efficiency of carrier HT. In any case, we consider that the CQD-IB absorbing material is sandwiched between two selective contacts, ESC and HSC, thus achieving the basic structure of the IBSC represented in Figure 1a. Additionally, when working under illumination, we also assume the following hypotheses.

1. Non-radiative recombination is neglected, based on the beneficial features of CQDs [7,8,9,10,11,12] mentioned in the first paragraph in Section 1.
2. The Fermi-level splits into three quasi-fermi levels, $E_{FC}$, $E_{FI}$, and $E_{FV}$, for describing the carrier concentration in the three bands.
3. Δn and Δp represent the carrier excess—with respect to the equilibrium ones, $n_{0}$ and $p_{0}$, respectively—in the CB (electrons) and VB (holes) of the IB-material [136]. These are just the bands that inject current density ($J_{e}\equiv J_{CB}$, $J_{h}\equiv J_{VB}$) via the selective contacts, ESC and HSC, the IB being isolated.
4. As no current is extracted from the IB, the net electron rate photo-excited from the VB to the IB (generation minus recombination, ${\left(g-r\right)}_{IV}$) must equal the one from the IB to the CB, ${\left(g-r\right)}_{CI}$, that is, ${\left(g-r\right)}_{IV}={\left(g-r\right)}_{CI}$.
5. The quasi-Fermi level for electrons in the IB is clamped at its equilibrium position to keep the IB half-filled with electrons. This can be achieved by heavily *n*-doped PbS CQD solid-state films as experimentally proved in [70,137].
6. The IBSC works under the low-injection approximation and, when carrier generation becomes uniform, then the electron and hole current densities $J_{e}\equiv J_{CB}$ and $J_{h}\equiv J_{VB}$ are described by a quasi-drift diffusion model, while that of the isolated IB, $J_{IB}$, becomes negligible [136]. Uniform carrier generation, as in SGSCs, can be accomplished by using light-trapping techniques, which randomize the light inside the material. Uniform recombination rates are reached as the diffusion length of the carriers involved (electrons in the CB, hole in the VB), $L_{e}$ and $L_{h}$, increase [136].

**Figure 2:**
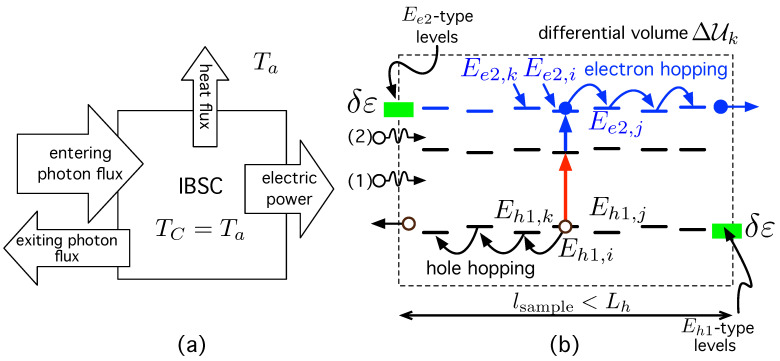
(**a**) The cell under illumination is an out-of-equilibrium system that absorbs (and emits) photons and partially converts their energy into electric power, a fraction being irreversible wasted in form of heat. The temperature of the cell is the same as that of the environment $T_{C}=T_{a}$. (**b**) Any of the differential volumes $\Delta U_{k}$ in which the cell is sub-divided. They can interchange electron/hole hopping particles and/or photons with others $\Delta U_{m}$.

To study carrier transport, we base our approach in the fact that, when operating, the IBSC is an out-of-equilibrium system. As represented in Figure 2a, the solar cell absorbs light and partially converts it into electric power. A part of the entering radiation energy is converted into heat, and another fraction is re-emitted as luminescent radiation—with non-zero chemical potential—at ambient temperature $T_{a}=T_{C}$. Our first step consists in dividing the out-of-equilibrium cell into sufficiently small volumes, $\Delta U_{k}$, so that they can be studied using quasi-equilibrium thermodynamics, but at the same time, these volumes are large enough so that the transport concepts make sense. We consider that the differential volume $\Delta U_{k}$ has a length $l_{\mathrm{sample}}\equiv \ell_{\Delta U}$ that is slightly shorter than the hole diffusion length, $L_{h}$, the average length it moves between generation and recombination [138,139,140]. This will be checked a posteriori in Section 3.

As illustrated in Figure 2b, once sub-band-gap photons (1) and (2) are absorbed inside the differential volume $\Delta U_{k}$, generating an extra electron-hole pair, the electron moves by hopping towards its selective contact (ESC) through $E_{e2}$-type levels, while the hole moves by hopping towards its selective contact (HSC) via $E_{h1}$-type levels.

With these hypotheses in mind, we are already at the point of being able to describe the carrier’s HT systems and the corresponding networks that represent them.

### 2.2. Model: Incoherent Electron and Hole Hopping Transport in CQD-IB Materials and Their Corresponding Networks

Let us consider, as a more general case, an array of disordered CQDs in which dots are randomly distributed in space. The dot density is $N_{D}$. For simplicity, any CQD *i* is described by three properties: the position vector $r_{i}$$\in R^{d}$ of its center (space embedding: d=2 in a film, d=3 in a three dimensional (3D) sample), its diameter $D_{QD,i}$, and a set of allowed energy levels (energy embedding: $\{E_{e2,i}$, $E_{e1,i}$, $E_{h1,i}\}$). Note that the subscript “*i*” is necessary because the energy levels can vary from one dot *i* to another *j* because of size fluctuations. Electrons with energy $E_{e2}$-type in Figure 2b are those that will give rise to the electron density current $J_{e}$, while holes with energy $E_{h1}$-type, will end up constituting $J_{h}$. Computing electron and hole hopping rates in our problem require the background that follows.

#### 2.2.1. Carrier Hopping Rates between Localized States *i* and *j* in a General Case

Carrier hopping is the dominant transport mechanism not only in disordered QCDs but also in organic disordered semiconductors [141,142], polycrystalline and amorphous semiconductors and, in general, in disordered solids [143]. The two most commonly used models for computing hopping rates between an occupied state and an unoccupied one are the Miller–Abrahams (MA) theory [133] and the Marcus model [144]. The latter is the most widely used in electron transfer involving electrochemical processes in molecular chemistry and biology [145,146,147,148,149]. The MA model is used in organic disordered semiconductors, polycrystalline and amorphous semiconductors, and many CQD-materials. We use the common MA model for CQD-materials [16,35,75].

The average rate transition for carriers between a localized state *i* with energy $\varepsilon_{i}$ and another one *j* with energy $\varepsilon_{j}$, $\Gamma_{ij}$, using detailed balance arguments [150], can be modeled using an MA-like hopping approach [133,141,142,150] as

(1) $$\Gamma_{ij}\approx \gamma_{0}\exp\left(-\beta_{ij}d_{E,ij}-\frac{\varepsilon_{ij}}{k_{B}T_{C}}\right),$$

where $\gamma_{0}$ is the attempt-to-escape frequency caused by phonons (or phonon frequency), $k_{B}$ is the Boltzmann constant, $T_{C}$ is the cell temperature, $d_{E,ij}$ is the (Euclidean) distance between the center of dots *i* and *j* (because of the space embedding in $R^{d}$), and $\beta_{ij}$ and $\varepsilon_{ij}$ are functions whose physical meaning is as follows. The first one, $\beta_{ij}$, is the tunneling decay between localized states *i* and *j*,

(2) $$\beta_{ij}={\left(\frac{2m_{B}^{*}\;\Delta E_{B_{ij}}}{\hbar^{2}}\right)}^{\frac{1}{2}},$$

where $m_{B}^{*}$ is the effective mass of the hopping particle (whatever it is, electron, hole, polaron, excition, etc.) in the barrier (B) material or in the ligand, $\Delta E_{B_{ij}}$ is the barrier height the quantum particle has to tunnel between states *i* and *j*, and *ℏ* is the reduced Planck constant. The second element in Equation (1), $\varepsilon_{ij}$, is a function that depends on the energy spacing between the involved states, $\varepsilon_{i}$, and $\varepsilon_{j}$, and on the energetic separation between them and the Fermi level $E_{F}$ [133,141,150],

(3) $$\varepsilon_{ij}=\frac{|\varepsilon_{i}-\varepsilon_{j}\left|+\right|\varepsilon_{i}-E_{F}\left|+\right|\varepsilon_{j}-E_{F}|}{2}.$$

The next step is to apply this tool to our problem. This is just the purpose of the following section.

#### 2.2.2. System: Electron and Hole Hopping Rates between Localized States in the CQD-IB Material

Let us start by establishing a notation to avoid making conceptual mistakes. We label the electron states with energy $E_{e2,i}$ (those with levels $E_{e2}$-type in Figure 2b) as “localized conduction states” (LCS). These are the (conduction) states between which electron hopping occurs, which will give rise to the electron flux injected into a load via the ESC. With the same argument, those states with energy $E_{e1,i}$ are called “localized intermediate states” (LIS), while $E_{h1,i}$, “localized valance states” (LVS). Note that in a perfectly ordered array of identical dots, the LCS, LIS, and LVS should become the CB, IB, and VB, respectively. In each of these bands, the carrier wave functions would ideally be delocalized over the whole volume of the IB material.

Once the notation has been established, the purpose of this section is to obtain the specific hopping rates between LCS and between LVS, respectively, since under the working hypotheses stated in Section 2, $J_{LIS}$ approaches zero. We focus thus on hopping rates between LCS and between LVS, respectively, which is important for characterizing the electron and hole current densities $J_{e}\equiv J_{CB}$ and $J_{h}\equiv J_{VB}$, as we have discussed in Hypothesis 6 in Section 2.

**Figure 3:**
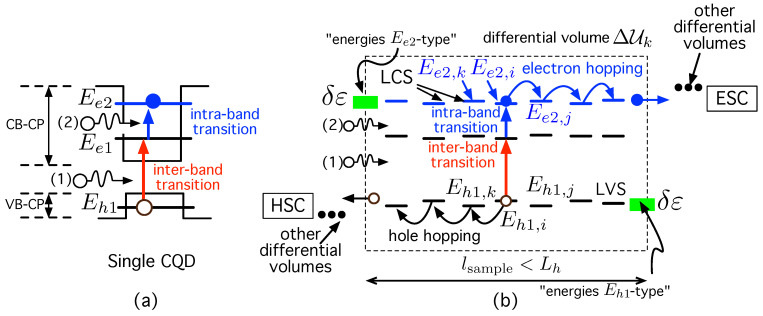
(**a**) Single CQD showing its CB and VB confinement potentials (CB-CP, VB-CP), respectively. $E_{e1}$ is the energy level associated to the 1S${}_{e}$ orbital, while $E_{e2}$ is the one associated to the 1P${}_{e}$ state. They are levels corresponding to electron states CB-CP. $E_{h1}$ stands for the first hole energy level in the VB-CP, 1S${}_{h}$ being its corresponding quantum state. (**b**) Any of the differential volumes $\Delta U_{k}$ in which the cell is sub-divided. Once sub-band-gap photons (1) and (2) are absorbed, generating an extra electron-hole pair, the electron moves by hopping towards its selective contact (ESC) through $E_{e2}$-type levels, while the hole moves by hopping towards its selective contact (HSC) via $E_{h1}$-type levels.

To do this, let us first focus on the single, isolated CQD represented in Figure 3a. For clarity, we have marked three energy levels that are of interest to the discussion that follows. $E_{e1}$ is the energy level associated to the 1S${}_{e}$ orbital, while $E_{e2}$ is the one associated to the 1P${}_{e}$ state. They are levels corresponding to electron states in the CB-CP. This is the reason why transitions between them are called “intra-band” or “inter-sub-band” transitions. In the VB-CP, there is a greater number of quantized holes because they have a higher effective mass than electrons. For illustrative purposes, we have labeled only the first of them. The reason is that, when we consider an array of CQDs, this level will be the one mainly involved in the transport processes. We have labeled this level $E_{h1}$, its corresponding quantum state being the orbital 1S${}_{h}$. A transition between 1S${}_{h}$ and 1S${}_{e}$ is called “inter-band” transition. These are also the orbitals considered in the experiments described in [70].

Let us again focus our attention on the whole disordered array of CQD that has a dot density $N_{D}$. In space, the dots are randomly distributed according to a uniform distribution (whose parameters will be specified in the simulation part of this work). In energy, some degree of the disorder appears, δε, caused by fluctuations in the size of the dots or even on their surfaces, as qualitatively illustrated (green rectangle) in Figure 3b. As a consequence of such a fluctuation, each of the interesting levels ($E_{e2}$, $E_{e1}$, and $E_{h1}$) changes its ideal delta-like DOS to a Gaussian DOS, with expected values $E_{e2}$, $E_{e1}$, and $E_{h1}$, respectively, and standard deviation σ,

(4) $$g_{1P_{e}}\equiv g_{E_{e2}}\left(E\right)=\frac{N_{D}}{\sigma_{}\sqrt{2\pi }}\exp\left(-\frac{{\left(E-E_{e2}\right)}^{2}}{2\sigma_{}^{2}}\right),$$

(5) $$g_{1S_{e}}\equiv g_{E_{e1}}\left(E\right)=\frac{N_{D}}{\sigma_{}\sqrt{2\pi }}\exp\left(-\frac{{\left(E-E_{e1}\right)}^{2}}{2\sigma_{}^{2}}\right),$$

(6) $$g_{1S_{h}}\equiv g_{E_{h1}}\left(E\right)=\frac{N_{D}}{\sigma_{}\sqrt{2\pi }}\exp\left(-\frac{{\left(E-E_{h1}\right)}^{2}}{2\sigma_{}^{2}}\right).$$

With this in mind, we now have the necessary tools and terminology to estimate the electron and hole hopping rates.

Let us first focus on an electron hopping between LCS, as the ones in Figure 3b. This is an electron hopping between states 1P${}_{e}$ in two different dots *i* and *j*. We use the notation $E_{e2,i}$ to refer to the energy level associated with the 1P${}_{e}$ state in QD *i*. These energy levels—$E_{e2}$-type levels in Figure 3b—takes values in the Gaussian DOS given by Equation (4). Using Equation (1), the electron hooping rate between two states 1P${}_{e}$ localized in different dots *i* and *j* is given by

(7) $$\Gamma_{ij}^{LCS}\approx \gamma_{0}\exp\left(-\beta_{ij}^{LCS}d_{E,ij}-\frac{\varepsilon_{ij}^{1P_{e}}}{k_{B}T_{C}}\right),$$

where $\beta_{ij}^{LCS}$ becomes into

(8) $$\beta_{ij}^{LCS}={\left(\frac{2m_{e,B}^{*}\;\Delta E_{B_{ij}}}{\hbar^{2}}\right)}^{\frac{1}{2}},$$

$m_{e,B}^{*}$ being the electron effective mass in the barrier material/ligand. The term $\varepsilon_{ij}^{1P_{e}}$ in Equation (7) is

(9) $$\varepsilon_{ij}^{1P_{e}}\approx \frac{|E_{e2,i}-E_{e2,j}|}{2},$$

since the IBSC is under low-level injection (Hypothesis (6) in Section 2).

Using the same arguments, the hole hopping rate between states 1S${}_{h}$ in two different CQD *i* and *k* will be

(10) $$\Gamma_{ik}^{LVS}\approx \gamma_{0}\exp\left(-\beta_{h}^{LVS}d_{E,ik}-\frac{\varepsilon_{ik}^{1S_{h}}}{k_{B}T_{C}}\right).$$

We are now in a position to define the network associated with electron transport between LCS and the network associated with hole transport involving LVS.

#### 2.2.3. Defining the Associated Networks for Study Electron and Hole Hopping Transport

As mentioned in Section 2, the IB-material comprises a set of CQDs with dot density $N_{D}$. Each dot *i* is defined by three parameters: the position vector $r_{i}$$\in R^{3}$ of its centre, its diameter ($D_{QD,i}$), and a set of allowed energy levels $\{E_{e2,i}$, $E_{e1,i}$, $E_{h1,i}\}$, which take values in the DOS defined by Equations (4)–(6).

To define the electron transport network, let us first focus on the electron hopping between states 1P${}_{e,i}$ and 1P${}_{e,j}$ with energy levels $E_{e2,i}$ and $E_{e2,j}$, respectively. We represent any state 1P${}_{e,i}$ as a node. We consider that an electron hopping between levels $E_{e2,i}$ and $E_{e2,j}$ is encoded by a link in the network of LCS.

The number of nodes (=1P${}_{e}$ states) available for an electron to hop is just the number of states 1P${}_{e}$ that is not occupied by an electron. This depends on the total number of levels $E_{e2}$-type (one per QD) and on the electrons excess in the CB,

(11) $$\Delta n=n_{0}\left(\exp\left(\frac{E_{FC}-E_{FI}}{k_{B}T_{C}}\right)-1\right),$$

with

(12) $$n_{0}=N_{C}\exp\left(\frac{-E_{L}}{k_{B}T_{C}}\right),$$

$N_{C}$ being the effective DOS in the CB, and $E_{L}\equiv G_{CI}$ the sub-gap between the CB and the IB. Thus, the number of available states for electron hopping in a differential volume $U_{k}$ is

(13) $$N_{HT}^{LCS}=\left(N_{D}-\Delta n\right)\cdot \Delta U_{k}\approx N_{D}\cdot \Delta U_{k}\equiv N,$$

because, under the hypothesis of low injection level, $\Delta n\ll N_{D}$ [136]. To lighten the notation, we will call $N_{HT}^{LCS}$ from now on as *N*, the number of nodes in the electron transport network.

At this point, we now need to introduce some NS concepts. The first one arises from the interaction between nodes (=states), that is, whether or not a carrier is allowed to hop from one node to another. In NS, when two nodes are directly connected by a link (by exchanging a charge carrier), then they are said to be adjacent or neighboring. The adjacency matrix A encodes whether or not there is a link ($a_{ij}=1$ or $a_{ij}=0$) between any two pairs of nodes *i* and *j*. A gives an idea of the structural connectivity of a network. Sometimes, this binary information is not enough, for example, if we want to study the dynamics of a carrier in the network. This requires quantifying the role or importance of any link by assigning each link a weight. In that case, the matrix is called weighted adjacency matrix W [107].

In our case, the weighted adjacency matrix corresponding to the electron hopping network with *N* nodes is an N×N matrix whose elements are

(14) $${\left(W\right)}_{ij}^{LCS}=\left\{\begin{array}{cc} 0 & ,ifi=i\\ \Gamma_{ij}^{LCS} & ,ifi\neq j, \end{array}\right.$$

being $\Gamma_{ij}^{LCS}$ given by Equation (7).

Once we have defined $W_{LCS}$ in Equation (14), we already have enough information to represent the electron LCS system as an electron hopping transport (eHT) network with the graph $G_{eHT}^{LCS}\equiv G\left(N_{}^{},M_{HT}^{LCS},W_{LCS}\right)$, where $M_{HT}^{LCS}$ is the number of links. Please, note that because of the method we have used to generate the links, the weighted adjacency matrix $W_{LCS}$ quantifies connections that have physical meaning according to hopping transport and explicitly includes the space-energy structure of the LCS system.

Using similar arguments for LVS, any 1S${}_{h,i}$ hole state with energy $E_{h1,i}$ is represented as a node (1S${}_{h,i}=$ node *i*), and a hole hopping between levels $E_{h,i}$ and $E_{h,j}$ is encoded by a link. As in the case of electrons, the corresponding network has, in a differential volume $U_{k}$, a number of nodes $N_{HT}^{LVS}=\left(N_{D}-\Delta p\right)\cdot \Delta U\approx N_{D}\cdot \Delta U\equiv N$, and a weighted adjacency matrix given by

(15) $${\left(W\right)}_{ij}^{LVS}=\left\{\begin{array}{cc} 0 & ,ifi=i\\ \Gamma_{ij}^{LVS} & ,ifi\neq j, \end{array}\right.$$

being $\Gamma_{ij}^{LVS}$ given by Equation (10).

The weighted adjacency matrix W for a given network contains enough information to study the motion of a particle (“walker”) on the network. This is just the purpose of the following section.

#### 2.2.4. Continuous Time Random Walks on Networks

In NS, once we have computed the weighted adjacency matrix W, we can obtain the so-called Laplacian matrix L [109,151], which in turn allows for studying the walker dynamics. To study carrier hopping between localized states, we use the CTRW tool [117]. The reason why we use random walks (RWs) is that the transport is incoherent due to carrier–phonon interactions (emission or absorption of phonons in each hop): the carrier loses its phase information every time it interacts [143].

The Laplacian matrix of any network is defined as [134]

(16) L=D−W,

where D is the diagonal degree matrix, whose elements $D_{i}=\sum_{i\neq j}{\left(W\right)}_{ij}$ are the strength or sum of the weights of all links directly connecting node *i* with the others. The Laplacian matrix allows for studying the time evolution of the probability $p_{kj}\left(t\right)$. This is the probability for a walker localized at node *j* (state or ket |j〉) to hop to another node *k* (=|k〉), $p_{kj}\left(t\right)$. As shown in [117,134,152], a walker performs a RW according to the master equation

(17) $$\frac{d}{dt}p_{kj}\left(t\right)=-\sum_{m}{\left(L\right)}_{km}p_{mj}\left(t\right).$$

whose formal solution is [117]

(18) $$p_{kj}\left(t\right)=\langle k|\exp\left(-Lt\right)|j\rangle =\sum_{n}\exp\left(-\lambda_{n}t\right)\langle k|q_{n}\rangle \langle q_{n}|j\rangle ,$$

$\lambda_{n}$ being the eigenvalues of L, which are real numbers and fulfill $\lambda_{n}\geq 0$. In this case, the RW associated with L is named edge-centric RW [134]. This means that any link in a node *i* is activated according to a process (the carrier hopping rate $\Gamma_{ij}$ in our problem). Once a link i↭j is activated, a random walker can use it to hop to the adjacent node *j*.

A useful related parameter that gives an idea of the global carrier hopping efficiency is the so-called average return probability (ARP) [117]

(19) $$\bar{p}\left(t\right)=\frac{1}{N}\sum_{j}p_{jj}\left(t\right)=\frac{1}{N}\sum_{j=1}^{N}\exp\left(-\lambda_{j}t\right).$$

High values of $\bar{p}\left(t\right)$ point out that the hopping is inefficient since the carrier tends to be localized at the initial node [153]. On the contrary, $\bar{p}\left(t\right)\ll 1$ means that the carrier, localized at any initial node in t=0, can quickly hop from node to node during the time interval *t*.

Finally, we label $L_{eHT}$ and $L_{hHT}$ the Laplacian matrices corresponding to the electron HT network and the hole HT network, obtained from Equations (14) and (15), respectively:

(20) $$L_{eHT}=D_{LCS}-W_{LCS},$$

(21) $$L_{hHT}=D_{LVS}-W_{LVS}.$$

These allow us to compute the ARPs of electrons and holes, respectively.

## 3. Results and Discussion

### 3.1. Methodology

As represented in Figure 3b, we have divided the out-of-equilibrium CQD-IBSC into sufficiently small volumes $\Delta U_{k}$ so that electron and hole HT can be studied before recombination occurs. We have considered that $\Delta U_{k}$ is a small volume in the shape of a cube, whose side has a length $l_{\mathrm{sample}}\equiv \ell_{\Delta U_{k}}<L_{h}$. And we have also mentioned in Section 2 that this working hypothesis was going to be demonstrated a posteriori. At this point, we already have all the tools for computing $L_{e}$ and $L_{h}$ and justifying the reason why is $L_{h}<L_{e}$. In this regard, the diffusion lengths for electrons and holes are [138,139]

(22) $$L_{e}=\sqrt{D_{e}\tau_{e}},$$

(23) $$L_{h}=\sqrt{D_{e}\tau_{h}}.$$

The first ingredient to estimate them is computing the diffusivities or diffusion coefficients,

(24) $$D_{e}=\frac{k_{B}T_{C}}{e}\;\mu_{e},$$

(25) $$D_{h}=\frac{k_{B}T_{C}}{e}\;\mu_{h},$$

which, in turn, depend on their respective mobilities, $\mu_{e}$ and $\mu_{h}$. We estimate them by using the Einstein–Smoluchowski relationship [86] as

(26) $$\mu_{e}\approx \frac{e}{k_{B}T_{C}}{\left({\bar{d}}_{E,ij}\right)}^{2}\;{\bar{\Gamma }}_{ij}^{LCS},$$

where ${\bar{d}}_{E,ij}$ and ${\bar{\Gamma }}_{ij}^{LCS}$ are the mean values (computed over the whole network) of the corresponding random variables $d_{E,ij}$ and $\Gamma_{ij}^{LCS}$ stated by Equation (7). Similarly, the hole mobility through localized states LVS is

(27) $$\mu_{h}\approx \frac{e}{k_{B}T_{C}}{\left({\bar{d}}_{E,ij}\right)}^{2}\;{\bar{\Gamma }}_{ij}^{LVS},$$

${\bar{\Gamma }}_{ij}^{LVS}$ being the mean value of $\Gamma_{ij}^{LVS}$ given by Equation (10). Once the mobilities have been estimated, we compute the diffusion coefficients, $D_{e}$ and $D_{h}$, by substituting Equations (26) and (27) into Equations (24) and (25):

(28) $$D_{e}\approx {\left({\bar{d}}_{E,ij}\right)}^{2}\;{\bar{\Gamma }}_{ij}^{LCS},$$

(29) $$D_{h}\approx {\left({\bar{d}}_{E,ij}\right)}^{2}\;{\bar{\Gamma }}_{ij}^{LVS}.$$

The second element to estimate the diffusion lengths stated by Equations (22) and (23) is computing the lifetime for electrons and holes. These can be estimated by using Roosbroek–Shockley-like relationships, as shown in [136],

(30) $$\frac{1}{\tau_{e}}\approx \frac{1}{n_{0}}\frac{8\pi }{h^{3}c^{2}}\int_{E}\alpha_{CI}E^{2}\exp\left(\frac{-E}{k_{B}T_{C}}\right)dE,$$

(31) $$\frac{1}{\tau_{h}}\approx \frac{1}{p_{0}}\frac{8\pi }{h^{3}c^{2}}\int_{E}\alpha_{IV}E^{2}\exp\left(\frac{-E}{k_{B}T_{C}}\right)dE,$$

where $n_{0}=N_{C}\exp\left(\frac{-E_{L}}{k_{B}T_{C}}\right)$ and $p_{0}=N_{V}\exp\left(\frac{-E_{H}}{k_{B}T_{C}}\right)$ are the electron and hole densities at equilibrium, $N_{C}$ and $N_{V}$ are the effective density of states in the CB and the VB, and $\alpha_{CI}$ and $\alpha_{IV}$ are the absorption coefficients involving the IB. These can be computed using the method explained in [154].

Finally, by substituting the diffusion coefficients (28)–(29) and the lifetimes from (30)–(31) into Equations (22) and (23), we can obtain the dependencies that $L_{e}$ and $L_{h}$ have, and estimate their values. Please note that these depend on the mean values of distances between dots and on the mean values of the corresponding carrier hopping rates.

Regarding this, in the effort to compute statistical values, we generate ensembles of networks with a sufficiently large number of networks. In the experiments carried out, we have found that it is sufficient to generate 50 realizations for each network. In any network realization, the dot centers are randomly distributed in a *d*-dimensional Euclidean space (d=2 for QDS films, and d=3 for CQD 3D samples), according to a uniform distribution U(a,b), with $a=2\times D_{QD}$ and $b=\ell_{\Delta U}=0.9\times L_{h}$. Any CQD *i* is represented by its position vector $r_{i}$$\in R^{d}$ (space embedding), its diameter ($D_{QD,i}$) and its set of allowed energy levels $\{E_{e2,i}$, $E_{e1,i}$, $E_{h1,i}\}$ (energy embedding). As the dot concentration in the IB-material is $N_{D}$, then the number of dots in the differential volume under study, $\Delta U_{k}={\left(L_{\mathrm{sample}}\right)}^{d}$, is $N_{D}\cdot \Delta U_{k}\equiv N$.

Table 1 lists the data used for the set of simulations that we describe below. Using the data listed in Table 1, we have obtained that $L_{h}\approx 200$ nm $<L_{e}\approx 316$ nm, which confirms our starting hypothesis.

### 3.2. Some Preliminary Considerations to Guide Carrier Transport Simulations in the Generated Networks

We have mentioned that the Laplacian matrices given by Equations (20) and (21) allows for computing the average return probability given by Equation (19). This provides an idea of the overall probability for a carrier to be localized at a dot or set of dots (or cluster). Thus, $\bar{p}\left(t\right)\to 1$ suggest very inefficient hopping because the carrier tends to be localized at the initial node [153], while $\bar{p}\left(t\right)\ll 1$ means that the carrier, initially localized at a given node in t=0, can speedily hop from node to node. The efficiency of the hopping transport can be characterized as [120]

(32) $$\eta_{HT}≐1-\bar{p}\left(t\right).$$

In any solar cell, it is of crucial importance to collect the electrons and holes as quickly as possible before they recombine. Aiming at increasing the efficiency of hopping transport, $\eta_{HT}$, we have several potential technological options. If we take a look at Equation (1) we could think of decreasing not only the inter-dot distance $d_{E,ij}$, but also $\beta_{ij}$ and $\varepsilon_{ij}$. It is worth discussing these points in the context of the IBSC conceptual framework:

1. Reducing the inter-dot distance $d_{E,ij}$ does not seem to have any drawback in principle: it would lead to a greater photon absorption per unit of volume/area and would also ease carrier transport. However, ref. [155] suggests that, at very small PbS-CQD radii, a relaxation of parity selection rules and a stronger electron-phonon coupling destroy the phonon bottleneck that helps electrons remain in excited states without thermalization. The study suggests that there could be an optimal dot size that, on the one hand, allows a sufficiently long energy separation between the ground ($E_{e1}$) and the excited state ($E_{e2}$), and on the other hand, maintains the phonon bottleneck effect, avoiding thus fast thermalization. This is essential for the IBSC concept since it requires to have three electron gases with their corresponding quasi-Fermi levels, as stated by Hypothesis (2) in Section 2.
2. In the effort of reducing the value of the tunneling decay $\beta_{ij}$ between localized states *i* and *j*—Equation (2)—we could try to decrease the value of the potential barrier $\Delta E_{B_{ij}}$ and/or the value of the effective mass of the hopping particle $m_{B}^{*}$. Decreasing the potential barrier $\Delta E_{B_{ij}}$, at first sight, would increase the probability for the carrier to hop to the nearby dot. However, there is a design restriction. The average barrier height $\bar{\Delta E_{B}}$ cannot be less than the energy variation δε—Figure 3b—caused by the standard deviation σ in the dot size distribution (as stated by Equations (4)–(6)). The reason is that if $\bar{\Delta E_{B}}$<δε then there will be many CQDs whose $E_{e2,i}$-type levels would be in the continuum. This would reduce the photon absorption causing transitions from the (localized) LIS with energy $E_{e1,i}$ to the (now extended) $E_{e2,i}$ at the energy continuum. That is, in the case of CQDs structures for IBSCs, it seems that what is good for photon absorption is not good for carrier transport and vice-versa.
3. The most obvious option, as pointed out in Section 1, is to reduce $\varepsilon_{ij}$ in Equation (3) by manufacturing CQD films that are as homogeneous as possible, both in the dots’ size and in the chemical composition of their surface. Although impressive advances are being made to obtain highly mono-dispersive samples (standard deviation, ∼3% [81]), there seems to be a physical limit that is difficult to overcome [30,156]. In our simulations, we have considered $\varepsilon_{ij}\in$$(0.1\times k_{B}T_{C}$, $0.2\times k_{B}T_{C}$).

With these considerations in mind, we have carried out a set of simulations that allow us to observe the behavior of hopping transport when we vary some of the parameters on which it depends.

#### 3.2.1. Influence of the Dot Density $\rho_{QD}$

**Figure 4:**
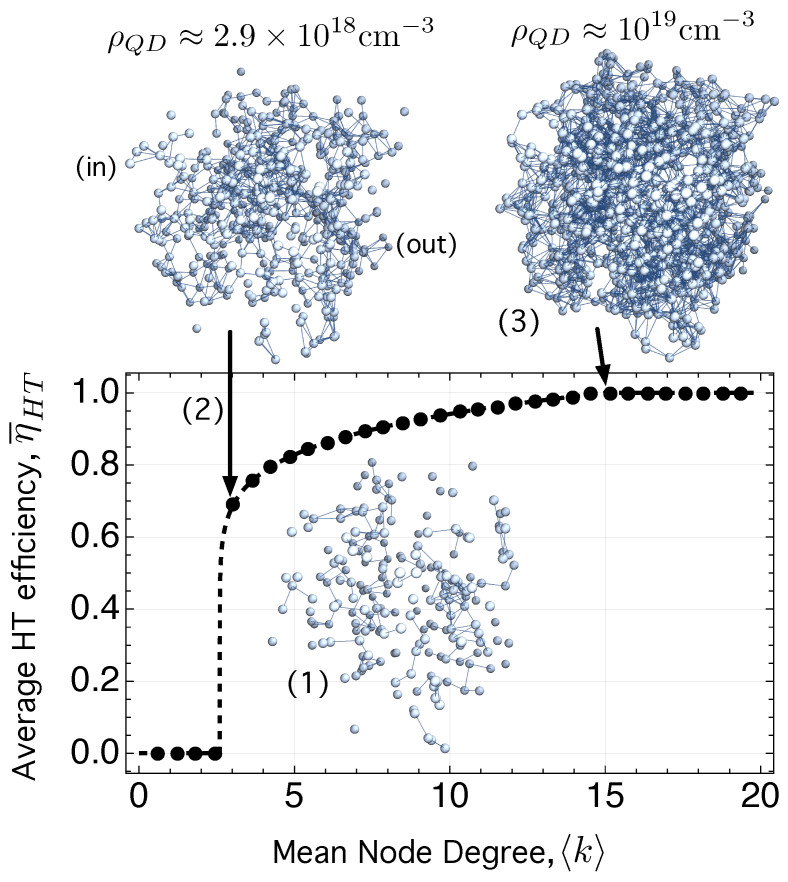
${\bar{\eta }}_{HT}$, average value of the electron hopping transport efficiency, $\eta_{HT}$, computed using Equation (32), as a function of the mean node degree 〈k〉. Each dot on the curve corresponds to the mean value obtained over 50 realizations of the network. Inset (1) shows a disconnected network with small isolated groups of dots. Inset (2) is a percolation sub-network connecting two opposite points in the sample, labeled “in” and “out”. Inset (3) represents a connected network in which all the nodes are linked to at least one other, allowing any carrier to hop across the network a distance on the order of the hole diffusion length, $L_{h}$.

We have mentioned in Section 1 that CQDs that are densely packed (∼$10^{19}$–$10^{20}$ cm${}^{-3}$) and heavily *n*-doped lead to a high absorption coefficient (∼$10^{4}$ cm${}^{-1}$) for the intra-band transition $E_{e1}\to E_{e2}$ [70], in the same order of magnitude than that of inter-band transitions $E_{h1}\to E_{e1}$. (See Figure 3a). Achieving a high QD density, $\rho_{QD}$, that is, a small inter-dot-distance, $d_{E,ij}$, is important not only for photon absorption but also for hopping transport and the extraction of the photo-generated carriers before recombination. Reducing $d_{E,ij}$ should increase $\Gamma_{ij}^{LCS}$ according to Equation (7). To illustrate its importance in electron hopping transport, we use Figure 4.

Figure 4 shows the average value (over 50 realizations of the network) of the electron hopping transport efficiency, ${\bar{\eta }}_{HT}$, as a function of the mean node degree 〈k〉. The mean node degree 〈k〉 quantifies in NS the average number of links (hops) between the nodes (states) of the e-HT network. When the density is small, the tunneling decay $\beta_{ij}^{LCS}$ in Equation (8) is not long enough to allow high enough $\Gamma_{ij}^{LCS}$ values (Equation (7). The consequence is that electrons can only make local hops between some nearest dots, which form small, interconnected groups of dots or clusters, as shown in the inset (1) of Figure 4. However, there is a value of the mean degree 〈k〉 for which one of the clusters becomes dominant and begins to grow to the detriment of the others. This cluster, called giant component (GC), has, in this particular case, the property of connecting two opposite points in the sample, labeled “in” and “out” in the inset (2) of Figure 4. The value 〈k〉=2.6 in Figure 4 is a critical point at which electron transport has an abrupt transition: ${\bar{\eta }}_{HT}$ changes suddenly from value 0 to value 0.62. It corresponds to the inset (2) in Figure 4, with a dot density $\rho_{QD}\approx 2.9\times 10^{18}$ cm${}^{-3}$.

This abrupt change is an example of a percolation transition, being ${\bar{\eta }}_{HT}$ its order parameter [157]. According to [157], which investigates the order parameter *m* in various situations (continuous, explosive, discontinuous, and hybrid percolation transitions), our network seems to have a hybrid percolation transition because it exhibits, at the same critical point ${\langle k\rangle }_{C}=2.6$, features of both first-order phase transitions (abrupt change in m= ${\bar{\eta }}_{HT}$) and second-order transitions (critical phenomena). The order parameter of our network, $m\equiv {\bar{\eta }}_{HT}$ fulfills

(33) $${\bar{\eta }}_{HT}\left(\langle k\rangle \right)\approx \left\{\begin{array}{cc} 0 & ,if\langle k\rangle <{\langle k\rangle }_{C}=2.6\\ 0.62\cdot {\left(\langle k\rangle -2.6\right)}^{0.15} & ,if\langle k\rangle \geq {\langle k\rangle }_{C}=2.6. \end{array}\right.$$

Note in Figure 4 that, for dot densities in the order $\rho_{QD}\approx 1$×$10^{19}$ cm${}^{-3}$, all nodes are connected to at least one other, which would allow any carrier to hop across the network a distance on the order of the hole diffusion length, $L_{h}$.

A similar result is obtained for the hole HT network.

**Figure 5:**
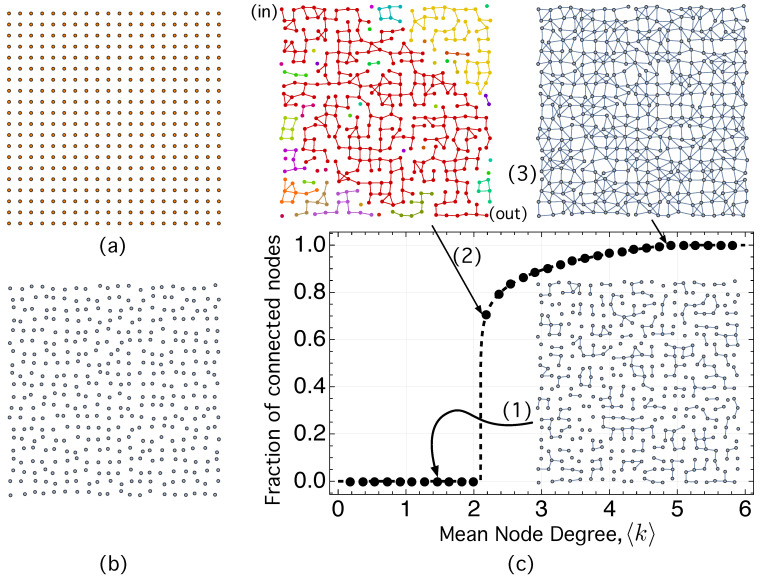
(**a**) Ideal ordered distribution of CQDs in a film. (**b**) The same distribution as before in which disorder has been introduced, as explained in the main text. (**c**) Fraction of connected nodes as a function of the mean node degree 〈k〉. The average barrier height $\bar{\Delta E_{B}}$ fulfills $\bar{\Delta E_{B}}$<δε to avoid degradation of transitions LIS→LCS. Insets (1–3) represent the different set of links connecting the nodes in (b) that emerge as the carrier effective mass is reduced. The reason why the link number increases when comparing one network with another is the reduction in $m_{B}^{*}$ when passing from inset (1) to (3). See the main text for further details.

These concepts are easier to visualize and understand in a CQD film since it is a two-dimensional structure (2D). Regarding this, Figure 5 will assist us in clarifying some previous concepts and presenting the framework of the simulations that follow.

#### 3.2.2. Influence of the Carrier Effective Mass in the Barrier/Ligand $m_{B}^{*}$

In particular, Figure 5a shows an ideal situation in which the CQDs are ordered, while Figure 5b represents the introduction of a small disorder in the position of the CDQs. In this case, the center of each CQD can deviate from its ideal position—in Figure 5a—by a distance 0.1 times the separation between neighbor dots, with a random angle between 0 and 2π.

Please note that, in Figure 5c, we consider the CQD distribution of Figure 5b as fixed, and we explore this structure the way in which links begin to appear as hopping increases. What is the phenomenon that increases hopping rates in these simulations? We have mentioned that, in the effort of increasing $\Gamma_{ij}$, it may not be a good idea to reduce, to a high extent, the height of the potential barrier, $\Delta E_{B_{ij}}$. The average barrier height $\bar{\Delta E_{B}}$ cannot be less than the energy variation δε—Figure 3b—because, when $\bar{\Delta E_{B}}$<δε, some CQDs have $E_{e2,i}$-type levels in the continuum, which reduces photon absorption. Thus, a feasible option for increasing hopping rate $\Gamma_{ij}$ consists in reducing $\beta_{ij}$ by replacing ligands/barrier materials with others in which the carrier has a lower effective mass $m_{B}^{*}$. This strategy has been adopted, for instance, in [35], in the context of solar cells based on PbS CQD solids. Ligands are exchanged by inorganic atomic ligands of tetrabutylammonium iodide (TBAI). The TBAI ligands assist in reducing the carrier’s effective mass and increasing its mobility. Moreover, in [82] ligand exchange has allowed for reducing the effective mass in CQDs of type HgSe/HgS (with $m_{e(HgSe)}=0.05m_{0}$ and $m_{e(HgS)}=0.03m_{0}$), considerably smaller than those in CdSe/CdS ($m_{e(CdSe)}=0.13m_{0}$ and $m_{e(CdS)}=0.21m_{0}$).

In this respect, we explore to what extent reducing the carrier effective mass $m_{B}^{*}$ increases $\Gamma_{ij}$. Figure 5c shows the fraction of connected nodes as a function of the mean node degree 〈k〉. The average barrier height $\bar{\Delta E_{B}}$ fulfills $\bar{\Delta E_{B}}$<δε to avoid degradation of transitions LIS→LCS. We have included different insets that aim to illustrate the progressive link appearance (i.e., carrier hopping between some dots occurs) as the effective mass in the ligand/barrier decreases. Please, note that in all of them, the position of the QDs is the same as in Figure 5b, the only difference being the existence of different links in each one of them. This is caused by the different effective masses in the ligand/barrier. The reason why the link number increases when comparing one network with another is the reduction in $m_{B}^{*}$ when passing from inset (1) to (3). This leads to a decrease in $\beta_{ij}$, an increase in $\Gamma_{ij}$, and, consequently, to the appearance of new links. For instance, inset (1) in Figure 5c, computed with a given $m_{B(1)}^{*}$ shows how small clusters appear, although still disconnected from each other. In contrast, inset (2), computed with a smaller $m_{B(2)}^{*}$$<m_{B(1)}^{*}$, illustrates the emergence of a GC (in red color) that provides several paths for carriers to hope from one side of the film to the opposite one. This component is also known as the minimum subnetwork (or “infinite cluster” in material science) or critical subnetwork for which a carrier in the node labeled “in” on the left side of the QD film can reach the opposite side at node “out”. In the network represented by inset (3) with $m_{B(3)}^{*}$$<m_{B(2)}^{*}$, all nodes are connected, forming a single network, in such a way that a random walker could travel through it.

#### 3.2.3. The Combined Influence of $\rho_{QD}$ and $m_{B}^{*}$

**Figure 6:**
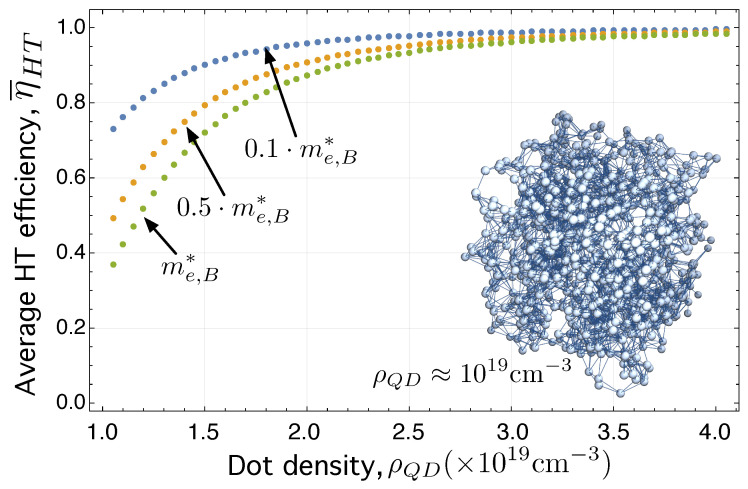
Average hopping transport efficiency (computed over 50 network realizations), ${\bar{\eta }}_{HT}$, for the electron HT network, $G_{eHT}^{LCS}$, as a function of the QD density, $\rho_{QD}$ (cm)${}^{-3}$, parametrized by different values of $m_{e,B}^{*}$, the electron effective mass in the barrier or in the ligand, depending on the nature of the colloidal quantum dot.

If we go back to a completely disordered 3D array of QCDs, like those in Figure 4, we can now compute the average value of the HT efficiency for different network sizes and investigate what is the influence of decreasing the value of the effective mass in the ligand or in the barrier material. Figure 6 represents the dependence of ${\bar{\eta }}_{HT}$ on the QD density, $\rho_{QD}$, parametrized by different values of $m_{e,B}^{*}$, in the particular case of the electron HT network, $G_{eHT}^{LCS}$.

**Figure 7:**
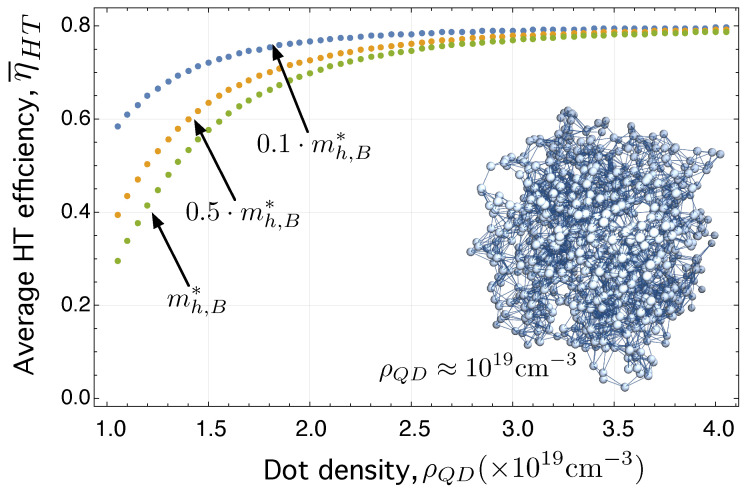
${\bar{\eta }}_{HT}$ of the hole HT network, $G_{eHT}^{LCS}$, as a function of the QD density, $\rho_{QD}$ (cm)${}^{-3}$, parametrized by different values of $m_{h,B}^{*}$.

Similarly, Figure 7 shows the average hopping transport efficiency (computed over 50 network realizations), ${\bar{\eta }}_{HT}$, as a function of the QD density, $\rho_{QD}$, but in this case for the hole HT network, $G_{hHT}^{LCS}$. The different dotted curves correspond to different values of the hole effective mass in the barrier or in the ligand, depending on the nature of the colloidal quantum dot, $m_{h,B}^{*}$.

Please note that, when comparing Figure 6 and Figure 7, it can be observed that the transport efficiency is worse in the case of the hole HT network $G_{hHT}^{LCS}$ than that in the electron HT network $G_{eHT}^{LCS}$. This is because their corresponding Laplacian matrices, $L_{eHT}$ and $L_{hHT}$, given by Equations (20) and (21) have different matrix elements. Specifically, the Laplacian for holes contains elements $\Gamma_{ik}^{LVS}$ that are smaller that those $\Gamma_{ij}^{LCS}$ in Equation (7). The reason is that $m_{h,B}^{*}>m_{e,B}^{*}$⇒$\beta_{ij}^{LVS}>\beta_{ij}^{LCS}$⇒$\Gamma_{ik}^{LVS}<\Gamma_{ij}^{LCS}$⇒${\bar{\eta }}_{hHT}$$<{\bar{\eta }}_{eHT}$.

#### 3.2.4. Some Final Considerations and Future Long-Term Work

We are aware that studying a system as extremely complex as an array of CQDs based on network tools—an alternative mathematical representation generated by selecting some of its properties (for example, the carrier hopping rates) without considering others—could be viewed as a reductionist approach [93,158]. We have shown in Section 1 that many works show that NS is a unifying, useful approach that helps study, within the same conceptual framework, a great variety of different systems whose elements interact among them. NS help capture the most essential properties of a system and, using its mathematical tools, makes it possible to explain and/or predict emergent phenomena, which go beyond the individual behavior of their constituent elements. NS is just only a complementary approach, which can be used in parallel with other well-established approaches. It does not intend and cannot replace the other successful methods that are applied in Materials Science and Nanotechnology. Related to this complementary and powerful character of the NS approach is the fact that a dense, close-packed ordered CQD-IB material could be modeled as a “multi-layer network” [159] with three layers. Each layer is, in turn, a network embedded in space and energy. Any of its nodes represents a quantum state of any of the three bands involved. In each layer, two nodes (quantum states) are linked if there is a carrier hop between them. We label these networks as CB-, IB-, and VB-networks. For instance, two nodes (states) in the CB network are linked if an electron hops between them. In turn, two nodes located in different layers (networks) are linked if there is an electron transition between them via photon emission/absorption. For instance, a node (state) in the IB network is linked with a node (state) in the CB network if the absorption or emission of a photon causes an electron transition between them. We denote a link between nodes in two different networks as“inter-link” to distinguish it from those that connect nodes in the same network (“intra-link”, or simply, link). This would allow for studying not only the carrier transport processes inside the layers but also the generation-recombination processes between layers. This seems, however, very difficult, long-term work.

## 4. Conclusions

This work has explored electron and hole hopping transport (HT) in colloidal quantum dot (CQD) intermediate band (IB) materials as complex networks that are embedded in both space and energy. Variations, even small, in the size of the dots, the chemistry of their surfaces, or their ligands produce some degree of energetic disorder δε. This causes electron hopping between localized conduction states (LCS) with different energy, $E_{e2,i}\to E_{e2,j}$, at CQDs *i* and *j*. Similarly, the disorder makes a hole hop between localized valence states (LVS) with different energy, $E_{h1,i}\to E_{h1,k}$, at CQDs *i* and *k*. The average carrier rate transition between two states *i* and *j* is modeled using Miller–Abrahams (MA) hopping rates $\Gamma_{ij}$, which are proportional to a negative exponential that includes both the spatial inter-dot distance $d_{E,ij}$ and an energy-difference function $\varepsilon_{ij}$ between the states involved. A CQD-IB material can be thus viewed as a complex system made up of a huge number of dots that interact with each other by exchanging charge carriers according to MA hopping rates $\Gamma_{ij}$. The essential idea when using Network Science is to map the system under study into a network (graph G) in which any interacting element is represented by a node (vertex) and the interaction between them by a link (edge). To do this, we have divided the CQD-IB material into small volumes $\Delta U_{k}$ so that electron and hole HT can be studied before recombination occurs. Indeed, electron and hole HT in $\Delta U_{k}$ occurs “inside” two different networks. The first one is the network formed by the LCS (=nodes) at different CQDs *i* and *j*, between which an electron hops ($E_{e2,i}\to E_{e2,j}$), forming a link according to the hopping rate $\Gamma_{ij}^{LCS}$. We have called this network electron HT network, $G_{eHT}^{LCS}$. The second transport network corresponds to LVS (=nodes) at different CQDs *i* and *k*, between which a hole hops ($E_{h1,i}\to E_{h1,k}$), appearing thus a link ruled by the hopping rate $\Gamma_{ik}^{LVS}$. We have called it the hole HT network, $G_{eHT}^{LCS}$. Each of the electron and hole HT networks defines a Laplacian matrix L that contains information about its corresponding hopping rates. The Laplacian matrix allows for studying the time evolution of the probability for a carrier (“walker”) localized at node *j* to hop to another node *k*. The average return probability (over the *N* network nodes), $\bar{p}\left(t\right)=\frac{1}{N}\sum_{j}p_{jj}\left(t\right)$ gives an idea of the global carrier hopping. $\bar{p}\left(t\right)\ll 1$ means that the carrier can quickly hop from node to node during the time interval *t*. If the efficiency of the hopping transport $\eta_{HT}≐1-\bar{p}\to 1$, then the carrier can quickly navigate most of the network. Aiming to obtain results that are statistically significant, each simulation is repeated a sufficiently large number of times. Thus, ${\bar{\eta }}_{HT}$ is the mean value of $\eta_{HT}$ over 50 realizations of a network. The simulations carried out have led to the following conclusions:

1. We have studied the influence of the dot density $\rho_{QD}$ on the average HT efficiency ${\bar{\eta }}_{HT}$. Achieving higher $\rho_{QD}$, or equivalently, smaller inter-dot-distance, $d_{E,ij}$, is important not only for increasing sub-band-gap photon absorption but also for enhancing electron and hole HT (and, thus, for the injection of the increased photo-generated current). Reducing $d_{E,ij}$ increases both electron and hole HT rates, $\Gamma_{ij}^{LCS}$ and $\Gamma_{ik}^{LVS}$. We have found that, as $\rho_{QD}$ increases, ${\bar{\eta }}_{HT}$ has a hybrid percolation transition at $\rho_{QD}\approx 2.9\times 10^{18}$ cm${}^{-3}$, in which ${\bar{\eta }}_{HT}$ changes abruptly from $\eta_{HT}\approx 0$ to $\eta_{HT}\approx 0.62$. For dot densities $\rho_{QD}\geq 1\times 10^{19}$ cm${}^{-3}$ all nodes are connected to at least one other, which allows any carrier to hop across the network a distance on the order of the hole diffusion length, $L_{h}$. Although the proposed model predicts the beneficial feature of increasing ${\bar{\eta }}_{HT}$ by means of reducing $d_{E,ij}$, however, special care should be taken. This is because, as suggested in [155], there may be an optimal dot size that, on the one hand, allows a sufficiently long energy separation between the ground and the excited state and, on the other hand, maintains the phonon bottleneck effect, avoiding thus fast thermalization. This is essential for the CQD-IB solar cell, which requires to have three electron gases with their corresponding quasi-Fermi levels.
2. We have also explored how carrier hopping rate can be increased by reducing the tunneling decay between two any localized states *i* and *j*, $\beta_{ij}={\left(2m_{B}^{*}\;\Delta E_{B_{ij}}/\hbar^{2}\right)}^{1/2}$. Prior to investigating the effect of the effective mass $m_{B}^{*}$, we have found that decreasing the average barrier height $\bar{\Delta E_{B}}$ has a design restriction. $\bar{\Delta E_{B}}$ cannot be smaller than the energy variation δε caused by the standard deviation σ in the dot size distribution. The reason is that if $\bar{\Delta E_{B}}$<δε then there will be CQDs whose $E_{e2,i}$-type levels would be in the continuum. This would reduce the photon absorption causing transitions from the (localized) LIS with energy $E_{e1,i}$ to the (now extended stated) $E_{e2,i}$ in the CB continuum.
3. By imposing the constraint $\bar{\Delta E_{B}}$<δε, we have studied the effect of reducing the effective mass in the ligand/barrier, $m_{B}^{*}$. For illustrative purposes, we first focused on a two-dimensional case that simulates a CQD film. Keeping the CQD positions constant, we have progressively reduced the $m_{B}^{*}$ value. Those networks that represent CQD films with smaller $m_{B}^{*}$ have more links because it is easier for the carrier to hop to the adjacent CQD. These results agree with those observed experimentally in the context of solar cells based on PbS CQD solids in which ligand exchange reduces $m_{B}^{*}$ and increases mobility [35].
4. Going one step further, we have carried out simulations to study the combined effect of increasing $\rho_{QD}$ and decreasing $m_{B}^{*}$, this time in samples of randomly distributed CQDs in a three-dimensional volume $\Delta U_{k}$. We have computed the average value of the HT efficiency ${\bar{\eta }}_{HT}$ as $\rho_{QD}$ increases, parametrized by decreasing $m_{B}^{*}$ values, for both the electron HT network, $G_{eHT}^{LCS}$, and the hole HT network $G_{hHT}^{LCS}$. The results point out that ${\bar{\eta }}_{HT}$ is smaller in the case of the hole HT network $G_{hHT}^{LCS}$ than in $G_{eHT}^{LCS}$. This is because the Laplacian for holes contains elements $\Gamma_{ik}^{LVS}$ that are smaller that those $\Gamma_{ij}^{LCS}$ in the Laplacian for electrons. In turn, this is because $m_{h,B}^{*}>m_{e,B}^{*}$⇒$\beta_{ij}^{LVS}>\beta_{ij}^{LCS}$⇒$\Gamma_{ik}^{LVS}<\Gamma_{ij}^{LCS}$⇒${\bar{\eta }}_{hHT}$$<{\bar{\eta }}_{eHT}$.

## Figures and Tables

**Table 1:** Data for the numerical example illustrated throughout this work.

Parameter	Symbol	Value
Density of quantum dots	$N_{D}$	Variable
Size of quantum dots	$D_{QD}$	4 nm
Effective density of states in the CB	$N_{C}$	$10^{18}$ cm${}^{-3}$
Effective density of states in the VB	$N_{V}$	$10^{18}$ cm${}^{-3}$
Absorption coefficient causing transitions IB → CV	$\alpha_{CI}$	$10^{4}$ cm${}^{-1}$
Absorption coefficient causing transitions VB → IB	$\alpha_{IV}$	$10^{4}$ cm${}^{-1}$
Electron lifetime	$\tau_{e}$	100 ps
Hole lifetime	$\tau_{h}$	50 ps
Electron diffusivity	$D_{e}$	10 cm${}^{2}$ s${}^{-1}$
Hole diffusivity	$D_{h}$	8 cm${}^{2}$ s${}^{-1}$
Electron mobility	$\mu_{e}$	0.25 cm${}^{2}$ V${}^{-1}$ s${}^{-1}$
Hole mobility	$\mu_{h}$	0.2 cm${}^{2}$ V${}^{-1}$ s${}^{-1}$
Electron diffusion length	$L_{e}$	316 nm
Hole diffusion length	$L_{h}$	200 nm

## Data Availability

Not applicable.
